# Individual Differences in Gambling Proneness among Rats and Common Marmosets: An Automated Choice Task

**DOI:** 10.1155/2014/927685

**Published:** 2014-05-27

**Authors:** Francesca Zoratto, Emma Sinclair, Arianna Manciocco, Augusto Vitale, Giovanni Laviola, Walter Adriani

**Affiliations:** ^1^Section of Behavioural Neuroscience, Department of Cell Biology and Neurosciences, Istituto Superiore di Sanità, Viale Regina Elena 299, 00161 Rome, Italy; ^2^Bambino Gesù Children's Hospital IRCCS, Rome, Italy; ^3^Istituto di Scienze e Tecnologie della Cognizione, National Research Council (ISTC-CNR), Rome, Italy

## Abstract

Interest is rising for animal modeling of pathological gambling. Using the operant probabilistic-delivery task (PDT), gambling proneness can be evaluated in laboratory animals. Drawing a comparison with rats, this study evaluated the common marmoset (*Callithrix jacchus*) using a PDT. By nose- or hand-poking, subjects learnt to prefer a large (LLL, 5-6 pellets) over a small (SS, 1-2 pellets) reward and, subsequently, the probability of occurrence of large-reward delivery was decreased progressively to very low levels (from 100% to 17% and 14%). As probability decreased, subjects showed a great versus little shift in preference from LLL to SS reinforcer. Hence, two distinct subpopulations (“non-gambler” versus “gambler”) were differentiated within each species. A proof of the model validity comes from marmosets' reaction to reward-delivery omission. Namely, depending on individual temperament (“gambler” versus “non-gambler”), they showed either persistence (i.e., inadequate pokes towards LLL) or restlessness (i.e., inadequate pokes towards SS), respectively. In conclusion, the marmoset could be a suitable model for preclinical gambling studies. Implementation of the PDT to species other than rats may be relevant for determining its external validity/generalizability and improving its face/construct validity.

## 1. Introduction

The emerging field of neuroeconomics is focused—by interdisciplinary approaches [1, 2]—on the ability of animals, including humans, to process multiple alternatives and to choose an optimal course of action. One of the major research areas in neuroeconomics is decision-making under risk and/or uncertainty (e.g., [3]). To know whether subjects will tend to seek or avoid risk under various circumstances (e.g., [4–7]) is mostly relevant for a number of economic activities, such as investment, speculation, and gambling (e.g., [8–10]).

Betting money represents a recreational activity for the majority of people, but it may become a serious, clinically relevant, behavioural disorder for others (DSM-IV-TR and DSM-V, [11–13]). Pathological gambling, which affects up to 5.3% of adult humans in western societies [14], is rapidly emerging as both a social and a health concern [15, 16]. Interest is therefore rising for animal modeling of gambling proneness. Indeed, evidence obtained on nonhuman subjects can inform the research on human pathological gambling in several ways (for a review, see [17]).

There has been increasing interest in the common marmoset (*Callithrix jacchus*, a species of New World monkeys) as a model for experiments in neuroscience. They have been used in different areas of biomedicine, including neurobiology [18, 19], toxicology [20, 21], and immunology [22, 23] and for the study of neurodegenerative disorders [24–28]. To our knowledge, there is only one study evaluating decision-making under uncertainty in common marmosets [10]. The task employed by these authors involved the choice between two bowls (containing constant or risky reward): caps of different colors were indication of a nonrisky or risky choice. Similar settings, employing bowls and colored caps, were used for most of the experiments in nonhuman primates [29]. Very little is known about the possibility to run these probabilistic reward tasks, in nonhuman primates, by means of automated, operant panels. The present study aims at evaluating the potential of marmosets tested by means of automated, operant panels as an animal model for human (pathological) gambling.

Gambling proneness can be evaluated in laboratory settings using the probabilistic-delivery task (PDT), an operant protocol classically performed in rats [30–38]. The PDT is based on the choice between a “Small & Sure” (SS) and a “Large & Luck-Linked” (LLL) reward [39, 40]. After a basal preference for LLL is established, the probability that large-reward delivery actually occurs decreases progressively to very low levels. Thus, to maximize the payoff, subjects should be flexible enough to abandon their large-reward preference, previously developed. Since optimal performance is expressed by a choice-shift towards a small reward, this entails a self-control effort in order to overcome the “innate drive” that justifies LLL attractiveness [35–37]. By contrast, a sustained preference for a large but extremely rarefied reward denotes temptation to gamble. The unrewarded visits to the poking holes, expressed during the postchoice timeout interval, can also be measured. Such inadequate responding can be considered an index of frustration (i.e., restlessness or persistence; see Methods) due to the punishment (consisting in reward-delivery omission). An experimental apparatus, originally developed for rodents [41], has been recently adapted to the common marmoset [42]. In such a recent experiment, we showed that impulsive behaviour can be reliably modeled in a delayed-reward setting.

**Figure 1:**
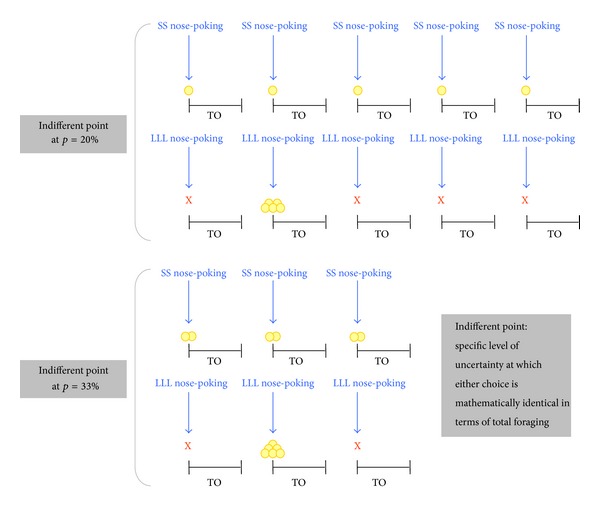
Schematic representation of the “indifferent” points. Arrows represent nose-poking tested under the PDT protocol: nose-poking in “Small & Sure” (SS) hole resulted in the certain delivery of 1-2 pellets, whereas nose-poking in “Large & Luck-Linked” (LLL) hole resulted in the delivery (or not) of 5-6 pellets, according to the level of probability “*p*,” which was decreased progressively over days. Thus, if the ratio between large and small reward size was 5-fold (upper part of the scheme), then the indifferent point was at *p* = 20%. By contrast, if the ratio between large and small reward size was 3-fold (lower part of the scheme), then the indifferent point was at *p* = 33%. TO: timeout.

A landmark in the PDT protocol is the “indifferent” point, that is, the specific level of uncertainty at which the animals can choose either option freely with no effect on the overall economic convenience [34]. As an example, if the ratio between large and small reward size was 3- to 5-fold (as in the present study), then the indifferent point (at which either choice was mathematically identical in terms of total foraging) ranged from *p* = 33% to *p* = 20%. This situation is depicted in Figure 1. We initially imposed a range of *P* values before the indifferent point (i.e., 100%, 50%) when LLL was always the optimal choice. Rats were then tested far beyond the indifferent point (i.e., 17%, 14%) when LLL became a suboptimal option and the economic benefit is attained unequivocally by choosing repeatedly the small-reward option.

This study aims (i) to evaluate marmosets as possible model for gambling proneness, using the PDT and drawing a comparison with rats, and (ii) to investigate interindividual differences as an approach to study the psychobiological bases and evolutionary roots of human gambling behaviour. Besides, the implementation of the PDT to species other than rats may be relevant for determining its external validity/generalizability and for improving its face/construct validity.

## 2. Materials and Methods

### 2.1. Ethical Note

All experimental procedures were approved by Institutional Animal Survey Board on behalf of the Italian Ministry of Health (licence to GL for rats and to AV for marmosets). Procedures were in close agreement with the European Communities Council Directive (86/609/EEC) as well as with Italian law (Italian Legislative Decree 116/92). As for marmosets, they were housed and cared for following the guidelines of both the Italian Association of Primatology and the International Primatological Society. All efforts were made to minimize animal suffering, to reduce the number of animals used, and to utilize alternatives to* in-vivo* techniques, if available.

### 2.2. Subjects and Housing

#### 2.2.1. Rats

Twelve adult (mean bodyweight 381.3 ± 9.5 g) Wistar male rats (Charles River, Italy) were housed in pairs inside Makrolon-type III cages with sawdust bedding, kept in an air-conditioned room (temperature 21 ± 1°C, relative humidity 60 ± 10%), on a 12 h reversed light-dark cycle (lights off at 7.00 a.m.). Water was available* ad libitum*, whereas food (Altromin-R, A. Rieper S.p.A., Vandoies, Italy) was available* ad libitum* until the start of the experimental protocol. Food restriction, imposed by the experimenter through a limited quantity of extra-food given at the end of each experimental session, was applied to increase the animal's motivation to work for food delivery (see below for details).

#### 2.2.2. Marmosets

Fifteen adult male and female common marmosets were involved in the present study. Each of the five family groups was housed in a home-cage measuring 80 × 130 × 220 cm. The floors of the cages were covered in wood shavings and each cage contained various forms of enrichment (including wooden branches, mobile objects, platforms, a wooden nest box, and other items) which were periodically changed. All families had auditory, olfactory, and partial visual contact with each other. One family at a time (on a daily rotation basis) was given access via tunnels to two other cages (experimental cages) of the same size as the home-cages, in an adjacent room. All rooms had a controlled temperature of 22 ± 1°C, a humidity of 50 ± 5%, and a 12-h light-dark cycle, with lights on at 6.00 a.m., which included exposure to UV-B lights. The diet consisted of specific commercial pellets for marmosets (Mucedola Ltd., Lecco, Italy), plus a portion of various fruits and vegetables. The monkeys were usually fed on a daily basis at approximately 9.00 a.m. whilst water and pellets were available* ad libitum*.

### 2.3. Apparatus

#### 2.3.1. Rats

Computer-controlled operant chambers, made of aluminium and Plexiglas with grid floor (Coulbourn Instruments, Allentown, PA, USA), were placed in an experimental room, adjacent to the animal room. The operant chambers were provided (on a same wall) with two nose-poking holes, two chamber lights (placed over each nose-poking hole), two feeder devices, and two food-magazines (each with a magazine light, signalling the length of the timeout, TO) where precision pellets (45 mg, F06555, Bio-Serv, Frenchtown, NJ, USA) were delivered. Nose-poking in either hole was detected by a photocell and was recorded by a computer (with custom-made software), which also controlled food delivery.

#### 2.3.2. Marmosets

Two computer-controlled operant panels (45 × 30 × 15 cm; “HOPs”, PRS Italia, Rome, Italy; [42]) were placed in each of the two experimental cages on a plywood platform (30 × 50 cm). The panels were provided with two hand-poking holes (one on each side), two purple hole lights above them, a single white house-light placed in the top middle of the panel, a feeder device, and two food-magazines (next to each hole with magazine lights) where precision pellets (45 mg, banana flavor, F0059, Bio-Serv, Frenchtown, NJ, USA) were delivered. The panels were connected through an interface to a computer, where software (“Sk020”, PRS Italia, Roma, Italy; [42]) controlled and recorded all events. Hand-poking in either hole resulted in the differential delivery of pellets in the corresponding food-magazine (see below for details).

### 2.4. Experimental Procedure

#### 2.4.1. Rats

After four weeks of habituation to the housing conditions and handling by the experimenters, rats were tested in the probabilistic-delivery (PD) task for gambling proneness, in the middle of the dark phase of their light-dark cycle (between 11.00 and 15.00 a.m.). Four chambers were used; each rat was tested daily in the same chamber at the same hour, five days a week. The total number of completed trials and the intertrial interval were not fixed, since rats were free to express nose-poking for food at their own, individually variable rate during the 25 min session.

After each daily session, rats were returned to their home-cage, where they were given an appropriate amount of standard food (approximately 4.5 g each) to keep their body weight at 90% of their free feeding body weight.

#### 2.4.2. Marmosets

The experimental sessions were performed between 9.00 and 13.00 a.m., five days a week. The total number of completed trials and the intertrial interval were not fixed, since marmosets were free to express hand-poking for food at their own, individually variable rate during the session. After the experimental session, in order to increase their motivation to work for food delivery on the following day, monkeys were fed at approximately 13.00 a.m., namely, after the experimental sessions had been completed, and received only small portions of fruit.

During the pretraining, training, and testing phases, two monkeys from the same family were tested simultaneously in the two opposite experimental cages, with their backs to each other. This was aimed to reduce the effects of social isolation. Before each session, panels remained covered with a wooden box until both subjects (selected to be tested) entered the experimental cages. At that point, the software was started and the wooden covers were removed. This was intended to prevent monkeys from interacting with the panels before they were turned on. Every day, at the end of the experimental sessions, the panels were again covered so that monkeys were free to move around the experimental cages without interacting with them.

Each session lasted 5 min, because the common marmoset has a relatively short attention span; attempts to use a longer testing time often made them loose interest in the apparatus.

### 2.5. Experimental Protocol

#### 2.5.1. Rats

*Training*. During the training phase (3 days), nose-poking in one of the two holes resulted in the delivery of 1 to 2 pellets in the corresponding magazine, whereas nose-poking in the other hole resulted in the delivery of 5 to 6 pellets in the other magazine. After nose-poking and before food delivery, the chamber light above the nose-poked hole was turned on for 4 s. Following food delivery, the corresponding magazine light was turned on for 15 s (timeout, TO), during which additional nose-poking was recorded but was without scheduled consequences (i.e., inadequate). The magazine light was then turned off, and the system was ready for the next trial. These training sessions allowed all subjects to reach a significant preference for the large reward.

*Testing*. During the testing phase (7 days) a probabilistic dimension was associated with the delivery of the large reward. The chamber lights were switched on after nose-poking following the usual schedule. However, delivery of the large reward was sometimes omitted, according to a given level of probability (*p* = percentage of actual food delivery over total demands). The small-reward delivery was unchanged. Hence, animals had a choice between a “Large & Luck-Linked” (LLL) or a “Small & Sure” (SS) reward. The probability level was kept fixed for each daily session and was decreased progressively over days (from *p* = 100% to *p* = 50%, *p* = 33%, *p* = 25%, *p* = 20%, *p* = 17%, and finally *p* = 14%).

#### 2.5.2. Marmosets

*Training*. Following familiarization and pretraining (see [42]), each monkey was placed individually in the experimental cage for the training phase (17 days). Hand-poking into one hole of the panel triggered the delivery of 1 to 2 pellets, whilst hand-poking into the other hole triggered the delivery of 5 to 6 pellets. The active session was indicated by the house light switched on (whilst the remaining lights were all off). After hand-poking, the house light was turned off and the purple hole light corresponding to the hand-poked hole was turned on for 2 s before food delivery. During and following food delivery, the corresponding magazine light was turned on for 6 s to signal the length of the timeout (TO), during which additional hand-poking was recorded but was without any scheduled consequences (i.e., inadequate). Then, the magazine light was turned off, the house light was turned on, and the system was ready for the next trial.

All monkeys but 2 (who had to be excluded from further testing for lack of interest in the apparatus) developed and displayed a preference for the large reward in this phase.

*Testing*. During the testing phase (21 days) a probabilistic dimension was associated with the delivery of the large reward. The purple hole lights were switched on after hand-poking following the usual schedule. However, delivery of the large reward was sometimes omitted, according to a given level of probability (“*p*” = percentage of actual food delivery over total demands). The small-reward delivery was unchanged. We intended to implement in monkeys the same rarefaction of large-reward delivery as in rats [34], with exactly the same progression except that three consecutive sessions were run for each level of “*p*.”

### 2.6. Analysis of Data and Experienced “Odds”

Three monkeys and one rat were excluded from data analysis as they failed to reach the inclusion criterion. The inclusion criterion (for both marmosets and rats) was defined as a preference for the large reward of more than 60% during the two sessions before the indifferent point [38–40, 42].

As a measure of gambling proneness, the dependent variable was the choice preference (%) for the LLL reward over total choices expressed. A sustained preference for the LLL reward may be an indication of gambling-prone behaviour [34–40]. As a general measure of motor impulsivity [42–45], the dependent variables were the average number of inadequate pokes per trial, calculated for each session, performed towards either the SS hole (i.e., restlessness) or the LLL hole (i.e., persistence). Restlessness values may be higher for subjects who, after poking into the LLL hole, start to ineffectively poke into the SS hole when the large-reward delivery is omitted. Such behaviour may be considered a motor consequence of an intolerance to uncertainty, namely, an index of motor impulsivity [42, 45]. On the contrary, persistence values may be higher for subjects who, after poking into the LLL hole, continue to ineffectively poke into the LLL hole when the large-reward delivery is omitted. Such behaviour may be considered an index of motor perseveration or cognitive inflexibility [46]. The following dependent variables were also considered: number of pellets earned per minute, number of trials completed per minute, and experienced odds.

Odds are defined as the mean number of omitted large-reward deliveries (because of “unlucky” events in the PDT) before a successful delivery (i.e., a “lucky” event in the PDT). The relation between “*p*” level and odds value is mathematical: odds = (1/*p*) − 1 or *p* = 1/(odds + 1). The present protocol employed a fully probabilistic generation of reward delivery versus omission, thus resulting in a totally random sequence of “lucky” versus “unlucky” trials. Therefore, a discrepancy likely appears between the “set” level of probability and the actually experienced rate of reinforcement, due to the stochastic fluctuations. Thus, for each session of the testing phase, we calculated the actually experienced “*p*” values for individual rats and marmosets (i.e., successful LLL/total LLL × 100), and turned them into the corresponding “experienced odds” values (i.e., (1/experienced probability) − 1). We plotted (on *y*-axis) the percent LLL preference, shown by rats and marmosets at each session, against (on *x*-axis) either (a) the set “*p*” values, selected by the experimenter, or (b) the “experienced odds,” once calculated. The latter was thus used to normalize percent LLL preference against an index of subjective impact of uncertainty. A logarithmic fit was also performed. Specifically, for each experimental rat or monkey, the slope of the preference-odds curve was calculated using Microsoft Excel functions, with Log (odds + 1) as *x*-axis and percent LLL choice as *y*-axis values.

On the basis of the median value of steepness of this preference-odds curve, we differentiated two distinct subpopulations [45, 47] in both rats and marmosets: a “non-gambler” one, which shifted quickly towards the SS hole (i.e., with a very steep slope), and a “gambler” one, with little or no shift. The median subject was assigned to the group to which its slope value was closest.

### 2.7. Statistical Analysis

Data were analyzed using repeated-measures analysis of variance (ANOVA). The general model was 2-level species × 2-level strategy × 7-level session, where species (rat* versus* marmoset) and strategy (the two subpopulations of gamblers* versus* non-gamblers) were a between-subject factor and session (the set probability per daily session) was a within-subject factor.

Statistical analysis was performed using Statview II (Abacus Concepts, CA, USA). Data are expressed as mean ± SEM. Significance level was set at *P* ≤ 0.05; ns = not significant; all statistics are two-tailed. Multiple comparisons were performed with Tukey's honestly significant difference (HSD) test.

## 3. Results

### 3.1. Choice Preference (%) for the Large-Uncertain Reward

On the whole, all animals showed a shift in preference towards the SS reward as the level of uncertainty increased. The ANOVA yielded a main effect of session (session: F(6,102) = 14.31, *P* < 0.001), confirming a progressive reduction of LLL preference when moving from *p* = 100% to *p* = 14% in probability of reward delivery.

**Figure 2:**
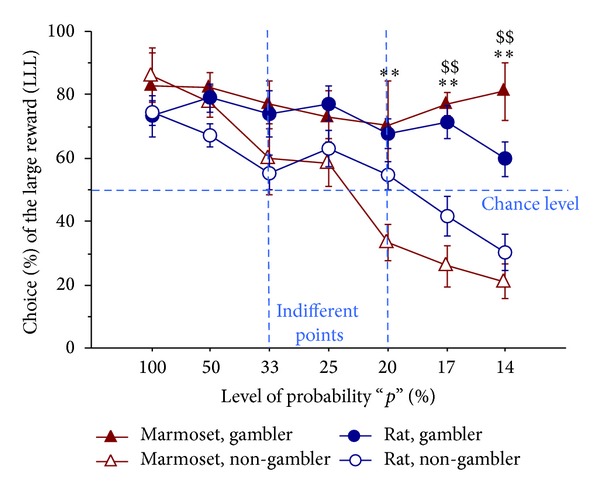
Gambling proneness: preference (%) for the LLL reward. Mean (±SEM) choice (%) of the large reward (LLL), shown by rats and marmosets belonging to the two distinct subpopulations (“gambler” versus “non-gambler”, *n* = 5-6 per group). On the final “gambling” part of the testing phase, “non-gambler” individuals (both rats and marmosets) were progressively shifting towards a clear-cut SS preference. By contrast, in “gamblers” (both rats and marmosets), LLL preference remained significant, even beyond the indifferent point (when LLL became a suboptimal option). **P < 0.01 gambler marmosets significantly different from non-gambler marmosets in post hoc test; ^$$^ *P* < 0.01 gambler rats significantly different from non-gambler rats in post hoc test.

As expected, individual differences in the preference for LLL versus SS rewards emerged in both rats and monkeys, with the identification of two distinct subpopulations (strategy: F(1,17) = 33.29, *P* < 0.001): one with a nearly horizontal curve (i.e., “gamblers”) and another with a very steep slope (i.e., “non-gamblers”). For both rats and marmosets, the “non-gamblers” showed a clear shift in preference from LLL to SS as the level of probability decreased. On the contrary, the “gamblers” maintained a significant attraction for LLL, even beyond the indifferent point, when LLL became a suboptimal option (strategy × session: F(6,102) = 7.47, *P* < 0.001; Figure 2).

Multiple post hoc analyses revealed a significant difference between “gambler” and “non-gambler” marmosets on the last 3 sessions (i.e., at *p* = 20%, 17%, 14%) and between “gambler” and “non-gambler” rats on the last 2 sessions (i.e., at *p* = 17%, 14%).

### 3.2. Experienced Odds

The ANOVA yielded a main effect of session (F(6,102) = 4.66, *P* < 0.001), confirming a progressive increase of odds values when moving from *p* = 100% to *p* = 14%. There were no main effects neither for strategy (F(1,17) = 0.16, *P* = 0.690 ns) nor for species (F(1,17) = 2.22, *P* = 0.154 ns) and no interactions as well (*P*s > 0.484). No difference emerged as well in multiple post hoc comparisons. This profile confirms no differences in odds values actually experienced by the four experimental groups.

The absence of any difference implies that a similar proportion of “lucky” versus “unlucky” pokes was experienced by subjects of the four groups. In other terms, neither group was “luckier” than the other. Thus, “gambler” individuals (both for marmosets and for rats) did not choose the LLL hole because simply they were “luckier,” but likely because of attraction to binge reward and/or insensitivity to its uncertainty. Vice versa, the “non-gamblers” preferred to shift towards the SS hole not because they were “unlucky” with LLL pokes, but likely because of sensitivity and hence aversion to uncertainty.

### 3.3. Number of Inadequate Pokes per Trial Towards the LLL Hole (i.e., Persistence)

The inadequate responding (i.e., pokes performed during the TO interval, without any scheduled consequence) measures the reaction of subjects to punishment (consisting in reward-delivery omission) and can be considered an index of frustration.

**Figure 3:**
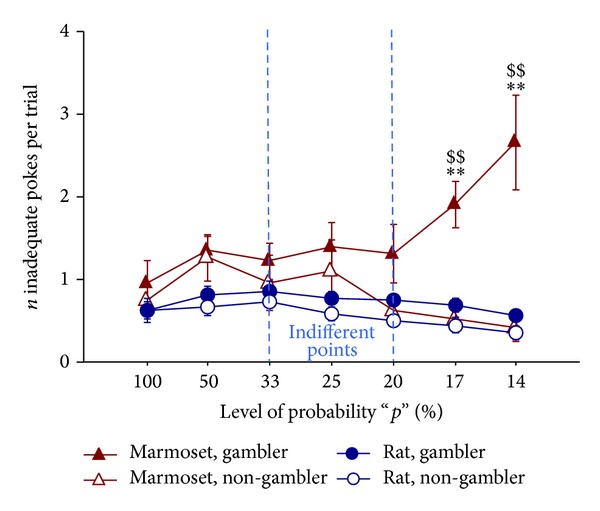
Persistence: number of inadequate pokes per trial towards LLL hole. Mean (±SEM) number per trial of LLL-inadequate pokes (i.e., performed during the postchoice TO interval, when they were without any consequence). Subjects are the same as in Figure 2. The “gambler” marmosets showed a significant increase in persistence at very low probability values, suggesting that they were still seeking for LLL during TO durations. **P < 0.01 gambler marmosets significantly different from non-gambler marmosets in post hoc test; ^$$^ *P* < 0.01 gambler marmosets significantly different from gambler rats in post hoc test.

At very low probability values, the “gambler” marmosets (but not rats) showed a significant increase in persistence, suggesting that they were still seeking for LLL during TO durations. This was reflected by significant interaction terms in the ANOVA (strategy: F(1,17) = 14.55, *P* = 0.001; species: F(1,17) = 20.39, *P* < 0.001; strategy × species: F(1,17) = 5.80, *P* = 0.028; strategy × session: F(6,102) = 5.21, *P* < 0.001; species × session: F(6,102) = 2.43, *P* = 0.031; species × strategy × session: F(6,102) = 4.09, *P* = 0.001, Figure 3). Accordingly, once the TO had elapsed, they maintained a clear expression in their choice for LLL, a notion that supports the gambling-like profile of these animals.

Multiple post hoc comparisons evidenced, on the last 2 sessions (i.e., at *p* = 17%, 14%), that the “gambler marmosets” were showing the highest levels of persistence. In fact, a significant difference was found between “gambler marmosets,” on one side, and “non-gambler marmosets” as well as “gambler rats,” on the other hand.

### 3.4. Number of Inadequate Pokes per Trial Towards the SS Hole (i.e., Restlessness)

Both rats and marmosets became overall more restless in the last sessions compared to the initial ones. Data are suggesting that, with progressive reward rarefaction, animals were increasingly disturbed by reward-delivery omission (session: F(6,102) = 19.14, *P* < 0.001).

**Figure 4:**
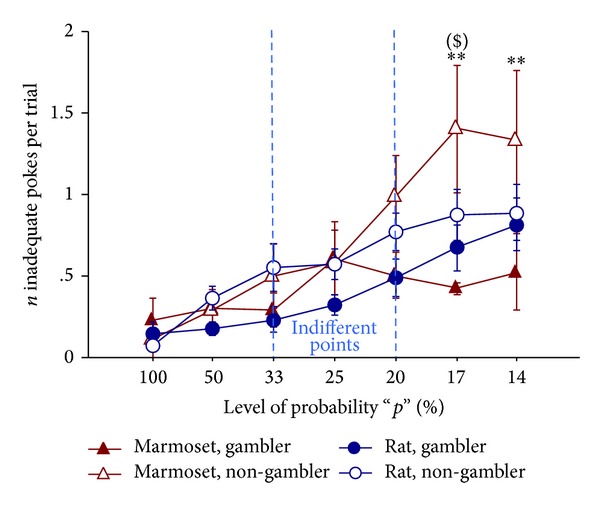
Restlessness: number of inadequate pokes per trial towards SS hole. Mean (±SEM) number per trial of SS-inadequate pokes (i.e., performed during the postchoice TO interval, when they were without any consequence). Subjects are the same as in Figure 2. While “non-gambler” marmosets showed a progressive increase of inadequate nose-pokes with decreasing levels of probability, “gambler” marmosets did not. Rats exhibited an intermediate profile. **P < 0.01 gambler marmosets significantly different from non-gambler marmosets in post hoc test; ^($)^0.05 < *P* < 0.1 non-gambler marmosets significantly different from non-gambler rats in post hoc test.

This profile was particularly evident in “non-gambler” marmosets, who showed a progressive increase of uncertainty-induced restlessness, with decreasing levels of probability. By contrast, such inadequate responding was remarkably low in “gambler” marmosets, suggesting that these animals are less sensitive to uncertainty and relatively unaffected by reward loss. This was reflected by significant interaction terms in the ANOVA (strategy: F(1,17) = 4.52, *P* = 0.048; strategy × session: F(6,102) = 3.33, *P* = 0.005; species × strategy × session: F(6,102) = 2.84, *P* = 0.014, Figure 4).

Multiple post hoc comparisons evidenced the “non-gambler marmosets” as showing the highest levels of uncertainty-induced restlessness. In fact, we found a significant difference between “non-gambler” and “gambler” marmosets on the last 2 sessions (i.e., at *p* = 17%, 14%) and, among the non-gambler subpopulation, a significant tendency (0.05 < *P* < 0.1) toward a difference between marmosets and rats on the penultimate session (i.e., at *p* = 17%).

### 3.5. Number of Trials Completed per Minute

**Figure 5:**
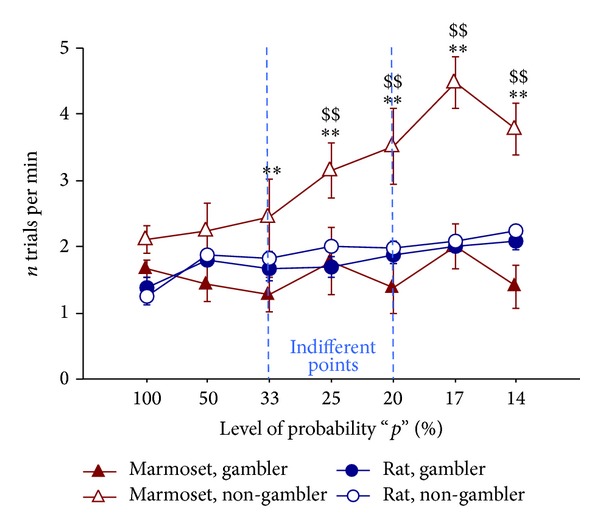
Trials per minute. Mean (±SEM) number of trials completed per minute. Subjects are the same as in Figure 2. A reaction to decreasing probability values was only shown by the “non-gambler marmosets,” who increased the number of trials completed per minute to compensate for reward-delivery omission. **P < 0.01 gambler marmosets significantly different from non-gambler marmosets in post hoc test; ^$$^ *P* < 0.01 non-gambler marmosets significantly different from non-gambler rats in post hoc test.

Interestingly, the number of trials completed per minute increased markedly as the level of probability decreased, but only in uncertainty-aversive marmosets. By contrast, “gambler” marmosets apparently did not adapt their reward-seeking pokes to balance for increasing reward omission. Specifically, the ANOVA yielded a main effect of strategy (F(1,17) = 15.96, *P* < 0.001), of species (F(1,17) = 5.69, *P* = 0.029), of session (F(6,102) = 13.83, *P* < 0.001), and their interactions (strategy × species: F(1,17) = 11.90, *P* = 0.003; strategy × session: F(6,102) = 4.74, *P* < 0.001; species × session: F(6,102) = 3.94, *P* = 0.001; species × strategy × session: F(6,102) = 3.81, *P* = 0.002, Figure 5).

Multiple post hoc comparisons confirmed that the “non-gambler marmosets” were completing the highest number of trials per minute. In fact, we evidenced a significant difference between “non-gambler” and “gambler” marmosets on nearly all sessions (i.e., at *p* = 33%, 25%, 20%, 17%, and 14%) and between marmosets and rats of the “non-gambler” subpopulation on all of the same sessions but the third (i.e., at *p* = 25%, 20%, 17%, 14%).

### 3.6. Number of Pellets Earned per Minute

The total number of pellets delivered per minute was significantly higher in “non-gambler marmosets” than in the remaining groups (strategy: F(1,17) = 19.80, *P* < 0.001; species: F(1,17) = 9.61, *P* = 0.006; strategy × species: F(1,17) = 10.40, *P* = 0.005). Pellets earned on average by “non-gambler marmosets” were 7.03 ± 0.47, compared with 3.57 ± 0.48 versus 3.62 ± 0.30 versus 4.17 ± 0.22 among “gambler marmosets,” “gambler rats,” and “non-gambler rats,” respectively. The amount of pellets obtained by “non-gambler marmosets” was particularly higher at very low probability values (session: F(6,102) = 41.40, *P* < 0.001; strategy × session: F(6,102) = 3.05, *P* = 0.009; species × session: F(6,102) = 5.80, *P* < 0.001, Table 1).

Multiple post hoc comparisons evidenced, on final sessions, a significant difference between “non-gambler marmosets” on one side and “gambler marmosets” as well as “non-gambler rats” on the other hand (at *p* = 20%, 17%, 14%).

## 4. Discussion

The search for the psychobiological bases and evolutionary roots of human gambling behaviour has exploited different nonhuman animal species in probabilistic reward tasks. In addition to rats, largely investigated in our lab [34–40], other species like pigeons and starlings, for example, have been studied extensively [5, 48–51]. Of special interest are the studies conducted so far on nonhuman primates: lemurs [29], capuchin monkeys [52, 53], rhesus monkeys [4, 6, 7], orangutans and gorillas [54], as well as chimpanzees and bonobos [54, 55], have been utilized.

The aetiology of pathological gambling is multifactorial; both genetic (e.g., polymorphisms in the genes that code for serotonin and/or dopamine receptors and transporters) [56–58] and socioenvironmental (e.g., [59, 60]) risk factors have been identified. Moreover, irrational beliefs and distorted erroneous perceptions are thought to play a key role. Indeed, cognitive theories of gambling behaviour propose that expectancies of winning, erroneous beliefs about the intervention of luck, illusions of control, and subsequent entrapment do contribute to the development and the maintenance of gambling patterns [61–63]. One of the cognitive distortions regarding the outcome of a stake, thought to specifically confer vulnerability, is the so-called “near-miss effect” (i.e., the experience of “almost winning” [64–66]). By means of a novel model of slot machine play (the “rodent Slot Machine Task,” rSMT), it has been recently demonstrated that rats are susceptible to this particular cognitive bias (i.e., putative-win signals in nonwinning trials [67]). Specifically, Winstanley and colleagues [67] found that (i) loss trials that resemble wins “near-misses” increased the behavioural expression of reward expectancy and that (ii) increased dopaminergic (DA) signalling (following administration of DA drugs) enhanced the expectation of reward delivery on loss trials. The latter may result from an inability to detect a negative prediction error (insensitivity to punishment) and/or from the generation of a positive reward expectancy [67].

The disruption of DA pathways significantly contributes to the propensity to gamble maladaptively [58]. With regard to the manifestation of the “near-miss effect,” the DA system is thought to be mostly involved because of its role in signalling reward expectancy. It may be also relevant to mention that prolonged exposure to dopamine replacement therapy induces pathological gambling in a minority of patients with Parkinson's disease (PD, e.g., [68–70]). Interestingly, a recent study found that PD patients with pathological gambling (compared to control PD patients) showed, in the ventral striatum, lower dopamine transporter (DAT) expression and increased synaptic dopamine levels [69]. Similarly, mice with a permanent reduction of DAT functioning (DAT knockdown) exhibited increased preference for riskier options in the mouse Iowa gambling task (IGT; [71]). However, a gambling-prone profile in the PDT was found in rats following lentivirus-mediated DAT overexpression in nucleus accumbens [36, 37].

In the present study, as classically observed in previous studies on rats [34–40], all marmosets showed a shift in preference from LLL to SS as the level of probability decreased. Gambling proneness can then be identified by the steepness of the preference-probability curve. Two distinct subpopulations were differentiated within each species: a “non-gambler” one, which shifted quickly towards SS, and a “gambler” one, with little shift. On one side, “non-gambler” rats and marmosets clearly showed optimal performance, preferring the smaller, certain reward and decreasing their preference for the large reward as it became more and more uncertain. On the other hand, “gambler” rats and marmosets maintained a relatively stable preference for the large reward, despite a decreasing probability of its actual delivery.

Many factors may explain the development of such a suboptimal preference for a binge but largely uncertain reward. One factor is hyposensitivity to risk, whereby the subjects are unable to foresee (as they should) an uncertainty in the outcome (usually, a source of aversion before choice) or to perceive the punishment of “losses” (represented by a randomly and frequently omitted delivery of reward). A second factor is habit-induced rigidity, whereby subjects seem to behave according to a strongly consolidated choice strategy. Such form of inflexibility may be due to a failure of negative reinforcement, namely, a lack of feedback-reaction to the uncertainty-induced aversion and/or to the omission-related punishment, just described [17, 46].

Another set of factors is hypersensitivity to rewards: the binge size of the reward has an excessive motivational impact over the subjects and monopolizes their attention, regardless of any other characteristic of the reward itself. There is also the possibility that the internal states, elicited by the risk of “loss” and experienced under conditions of uncertainty, become attractive as a secondary, conditioned feature. This is because the large reward (which sooner or later is eventually delivered) may well be generating an overwhelming peak of positive reinforcement. Similarly, all the surrounding signals and cues, that accompany and predict the features of uncertainty, may themselves become secondary rewarding stimuli. Regardless of which of these factors prevails in the PDT, the suboptimal preference for a large, rarefied reward is considered an index of “gambling-proneness” [17, 46].

The inadequate responding (i.e., pokes performed during the TO interval, without any scheduled consequence) allows to evaluate the reaction of subjects to the punishment (consisting in reward-delivery omission). It should be noted that inadequate pokes are mainly performed during the postchoice TO interval that follows an “unlucky” poke into the LLL hole (when animals have no pellets to eat) and can be considered an index of frustration [38, 72]. Compared to rats, marmosets' reaction to reward-delivery omissions showed some interesting peculiarities; namely, depending on individual temperament (“gambler” versus “non-gambler”), they showed either persistence (i.e., inadequate pokes towards the LLL hole) or restlessness (i.e., inadequate pokes towards the SS hole), respectively. The “non-gambler” marmosets showed, with increasing levels of LLL rarefaction, a progressive increase of uncertainty-induced restlessness and intolerance. This result, suggesting that they were disturbed by frequent reward-delivery omission, is in agreement with their “uncertainty-averse” profile. By contrast, such inadequate, restless responding was remarkably low in “gambler” marmosets, suggesting these animals to be less sensitive to uncertainty and/or relatively unaffected by reward loss. Instead, the “gambler” marmosets showed a significant increase in persistence at very low probability values, suggesting that they were still seeking for LLL during TO durations which followed each omission. Accordingly, they maintained their choice for LLL, which was then expressed once the TO had elapsed, a notion that supports the gambling-like profile of these animals. Interestingly, we reported similar results in rats about the localization of inadequate nose-pokes [39]. We found that, during the final “gambling” part (i.e., sessions beyond the indifferent point), “gambler” rats performed inadequate nose-pokes mainly towards LLL hole. With progressive reward rarefaction, these animals were still seeking for the LLL reward during TO durations (and persisted in choosing the LLL hole once the TO had elapsed).

The lack of omission-induced frustration in “gambler” marmosets and rats may be related to the effectiveness of magazine lights as a secondary reinforcer. In fact, these lights were turned on even when delivery of the large reward was omitted. It may be proposed that reward omission was not properly perceived as punishment by these animals, in that the light cue alone could sustain choice behaviour. Magazine lights turned on in the absence of food could have a much higher, secondary reinforcing value, triggering an anticipated drive for bingeing and persistent LLL seeking. Like in second-order schedules [73], this cue-induced secondary reward may sustain continued responding in the LLL hole, even though this implies a decreased overall foraging in the long term [34, 36, 38].

The “non-gambler” marmosets were able to obtain a considerably higher amount of pellets compared to “gambler” marmosets and to both “gambler” and “non-gambler” rats. This interesting finding is consequent to two independent phenomena: (i) they were able to make the “optimal choice” (i.e., choosing LLL before the indifferent point and SS during the final “gambling” part) and (ii) they progressively increased the number of completed trials, which compensated for the diminished gain associated with SS choice. Such a combination of these two phenomena was never evidenced in “non-gambler” rats during previous studies. Since the marmosets are primates and have well-developed prefrontal cortical areas in comparison with rodents [74], future studies using marmosets would be helpful to analyze neuronal mechanisms underlying the gambling attitude [10].

The results we obtained indicate that the common marmoset can be a suitable model for studies on decision-making under conditions of high uncertainty. A proof of the model validity comes from marmosets' individual reaction to reward-delivery omission. We report a clear-cut dissociation in inadequate responding depending on individual temperament (“gambler” versus “non-gambler”). This seems to resemble what has been reported in the clinical literature: while normal people are likely to modify their own seeking behaviour depending on reward outcome, human pathological gamblers are relatively unaffected by losses, hence persisting in this payoff-seeking activity despite repeated losses [75–78].

Nonhuman primate species differ markedly in their risk preferences: chimpanzees and orangutans are risk-seeking whilst bonobos and lemurs are risk-averse [29, 54, 55]. Although these differences can possibly be explained in terms of feeding ecology, it should be considered that the different risk preferences obtained in nonhuman primate studies are likely due to individual differences (for a review see [17]). Similarly, risk attitude in human behaviour is usually categorized into three types: risk-aversive, risk-prone, and risk-neutral (for a recent review, see [79]). Marked interindividual differences have been already reported in the common marmosets for a number of behavioural domains [80–82]. Furthermore, two recent studies evidenced clear-cut individual differences in decision-making under uncertainty in both rhesus macaques and marmosets ([7, 10]). As for rhesus macaques, it is known that risk sensitivity appears to be partly determined by the serotonergic system: (i) serotonin depletion increases risk proneness [83], a finding consistent with recent rodent data [38]; (ii) a length polymorphism in the gene that codes for serotonin transporter has a role in relation to intraspecific behavioural variability [84]. The aim of future studies will be to further characterize the role played by interindividual variations, by investigating marmosets' genetic profile (with particular reference to polymorphisms in the genes for serotonin receptors and transporter; [85]) and drawing correlations with traits of gambling proneness/aversion.

The setting used in the present study (i.e., operant panels placed inside experimental cages) has the potential to be adapted and used in more extensive ways, for permanent monitoring of subjects' operant-choices and spontaneous (social and nonsocial) behaviour. Such an automated social home-cage system would allow long-term, continuous data collection, which may provide a larger, more accurate picture of gambling-prone behaviour in these species. These systems, in which animals can freely move, interact with each other, and voluntarily access the operant panels, are promising for developing tasks in a more naturalistic environment, with increased ecological validity [17, 86].

## Figures and Tables

**Table 1:** Pellets per minute.

	*p* = 17%	*p* = 14%
Marmoset, gambler	2.69 ± 0.37	1.23 ± 0.66
Marmoset, non-gambler	7.84 ± 0.91	6.91 ± 0.89
Rat, gambler	2.79 ± 0.27	2.54 ± 0.32
Rat, non-gambler	3.15 ± 0.19	3.73 ± 0.26

Mean (±SEM) number of pellets earned per minute. Subjects are the same as in Figure 2. On final sessions at very low probability values (*p* = 17%, 14%), the amount of pellets obtained by “non-gambler” marmosets was considerably higher compared to “gambler” marmosets and rats of both subpopulations.
